# Recombinant Expression of Margatoxin and Agitoxin-2 in *Pichia pastoris*: An Efficient Method for Production of K_V_1.3 Channel Blockers

**DOI:** 10.1371/journal.pone.0052965

**Published:** 2012-12-26

**Authors:** Raveendra Anangi, Shyny Koshy, Redwan Huq, Christine Beeton, Woei-Jer Chuang, Glenn F. King

**Affiliations:** 1 Institute for Molecular Bioscience, The University of Queensland, St. Lucia, Queensland, Australia; 2 Department of Molecular Physiology and Biophysics, Baylor College of Medicine, Houston, Texas, United States of America; 3 Department of Biochemistry and Molecular Biology, Institute of Basic Medical Sciences, National Cheng Kung University College of Medicine, Tainan, Taiwan; University of Waterloo, Canada

## Abstract

The K_v_1.3 voltage-gated potassium channel regulates membrane potential and calcium signaling in human effector memory T cells that are key mediators of autoimmune diseases such as multiple sclerosis, type 1 diabetes, and rheumatoid arthritis. Thus, subtype-specific K_v_1.3 blockers have potential for treatment of autoimmune diseases. Several K_v_1.3 channel blockers have been characterized from scorpion venom, all of which have an α/β scaffold stabilized by 3–4 intramolecular disulfide bridges. Chemical synthesis is commonly used for producing these disulfide-rich peptides but this approach is time consuming and not cost effective for production of mutants, fusion proteins, fluorescently tagged toxins, or isotopically labelled peptides for NMR studies. Recombinant production of K_v_1.3 blockers in the cytoplasm of *E. coli* generally necessitates oxidative refolding of the peptides in order to form their native disulfide architecture. An alternative approach that avoids the need for refolding is expression of peptides in the periplasm of *E. coli* but this often produces low yields. Thus, we developed an efficient *Pichia pastoris* expression system for production of K_v_1.3 blockers using margatoxin (MgTx) and agitoxin-2 (AgTx2) as prototypic examples. The *Pichia* system enabled these toxins to be obtained in high yield (12–18 mg/L). NMR experiments revealed that the recombinant toxins adopt their native fold without the need for refolding, and electrophysiological recordings demonstrated that they are almost equipotent with the native toxins in blocking K_V_1.3 (IC_50_ values of 201±39 pM and 97±3 pM for recombinant AgTx2 and MgTx, respectively). Furthermore, both recombinant toxins inhibited T-lymphocyte proliferation. A MgTx mutant in which the key pharmacophore residue K28 was mutated to alanine was ineffective at blocking K_V_1.3 and it failed to inhibit T-lymphocyte proliferation. Thus, the approach described here provides an efficient method of producing toxin mutants with a view to engineering K_v_1.3 blockers with therapeutic potential.

## Introduction

Voltage-gated potassium (K_V_) channels are expressed in a wide range of cell types and tissues where they play key roles in physiological processes such as cell excitability, muscle contraction, and regulation of cardiac function [1]. K_V_ channels are composed of four α subunits that together form a functional channel [2]. There are nine subfamilies of K_V_ channels, with K_V_1.3 being one of eight subtypes in the K_V_1.x subfamily. K_V_1.3 channels are strongly upregulated during the activation of human effector memory T (T_EM_) cells, which play a crucial role in autoimmune diseases such as multiple sclerosis (MS), type-1 diabetes (T1D), and rheumatoid arthritis. The K_V_1.3 channel has consequently become a target for drugs to treat autoimmune diseases [3]–[8]. ShK, a sea anemone peptide that potently and selectively blocks K_v_1.3, was shown to be effective in six animal models of autoimmune disease: MS, T1D, rheumatoid arthritis, allergic contact dermatitis, bone resorption and delayed type hypersensitivity [9]. ShK will soon enter Phase 1 clinical trials for treatment of autoimmune disease [10].

Peptides derived from animal venom are the largest source of ion channel blockers and they have proved to be a valuable resource for developing drugs to treat a variety of diseases [10], [11]. Numerous peptidic K_V_1.3 blockers have been isolated from scorpion venom [12], [13], with members of the α-KTx subfamily of scorpion toxins displaying an extraordinary ability to distinguish between the large family of K_V_ channels and the maxi-K channel [14], [15]. Peptides from the α-KTx subfamily contain 23–43 amino acid residues and they share a common structural motif comprising an α-helix and 2–3 antiparallel β-strands stabilized by 3–4 disulfide bridges.

Although α-KTx peptides inhibit K_V_1.3 at sub-nanomolar concentrations, they often also inhibit other K_v_1.x subtypes. Thus, in order for these toxins to have therapeutic application, their selectivity needs to be engineered to avoid the deleterious effects caused by off-target activity on other K_V_ subtypes. However, progress in this area has been slow as isolation of toxins from crude venom yields minute amounts of material and chemical synthesis is time consuming and not cost-effective for production of mutant toxins for structure-activity relationship (SAR) studies. Recombinant production of K_V_1.3 blockers in the cytoplasm of *E. coli* generally necessitates oxidative refolding of the peptides in order to form their native disulfide architecture [16]. An alternative approach that generally avoids the need for refolding is expression of peptides in the periplasm of *E. coli* [17]–[19] but this often results in low yields. Thus, it would be highly beneficial to develop an efficient and cost-effective method for production of these potent ion channel modulators.

**Figure 1:**
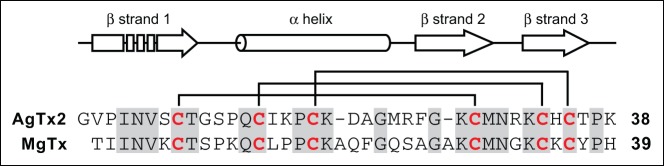
Alignment of the primary structures of agitoxin-2 (AgTx2) and margatoxin (MgTx). Conserved residues are highlighted with a grey background and cysteine residues are shown in red. The lines above the sequences indicate disulfide bonds. The secondary structure of AgTx2 (based on PDB file 1AGT) is shown above the sequences.

We have developed an efficient *Pichia pastoris* expression system for production of peptidic K_v_1.3 channel blockers. We demonstrate the efficacy of this system via the production of recombinant agitoxin-2 (rAgTx2) and margatoxin (rMgTx), K_V_1.3 blockers isolated from the venom of the scorpions *Centruroides margaritatus* (Central American bark scorpion) and *Leiurus quinquestriatus hebraeus* (Israeli yellow scorpion), respectively. These peptides are 46% identical at the amino acid level (Fig. 1). The 3D structures of AgTx2 (pdb: 1AGT) and MgTx (pdb: 1MTX) contain an α-helix anchored by three antiparallel β-strands stabilized by three disulfide bonds (Fig. 1) [20], [21]. Both peptides were previously produced by overexpression in *E. coli* but, although the recombinant peptides were fully functional, the yields of recombinant MgTx and AgTx2 were less than 2 mg/L [20]–[22].

*P. pastoris* has proved to be an excellent host for heterologous expression of disulfide-rich peptides [23]–[25]. In the present study we show that both MgTx and AgTx2 can be produced in *P. pastoris* with yields 6–8-fold higher than previously reported for production in *E. coli*. The recombinant AgTx2 and MgTx produced in *P. pastoris* show high potency towards mouse K_V_1.3 channels stably expressed in L929 mouse fibroblast cells and they also inhibited proliferation of human T-lymphocytes.

## Materials and Methods

### Ethics statement

All experiments involving live animals were performed in accordance with protocols approved by the Institutional Animal Care and Use Committee of Baylor College of Medicine.

### Production of recombinant MgTx, AgTx2, and a K28A mutant of MgTx

Expression and purification of MgTx, AgTx2, and a K28A mutant of MgTx were performed using the protocols we described previously [25]. Synthetic genes encoding MgTx and AgTx2, with codons optimized for *P. pastoris*, were constructed by ligating four complementary and overlapping oligonucleotides coding for the protein sequences of MgTx (UniProt P40755) and AgTx2 (UniProt P46111). The MgTx gene was amplified using PCR with the sense primer:

5′-GAATTC **CATCATCATCATCATCAT**ACTATTATTAACGTTAAGTGTAC-3′ that has an *Eco* R1 recognition site (underlined) and encodes six histidine residues (bold) to facilitate toxin purification. The antisense primer was:

5′-CCGCGG **TTA**ATGTGGGTAACACTTACACTTACCGTTCATACACTT-3′ with a *Sac* II recognition site (underlined) and a TTA stop codon (bold). The AgTx2 gene was amplified using PCR with the sense primer:

5′-GAATTC **CATCATCATCATCATCAT**GGTGTTCCAATTAATGTTTCTTGT-3′ that has an *EcoRI* recognition site (underlined) and encodes six histidine residues (bold) to facilitate toxin purification. The antisense primer was:

5′-CCGCGG **TTA**CTTTGGAGTACAATGACACTTTCTATTCAT ACACTT-3′ with a *SacII* recognition site (underlined) and a TTA stop codon (bold). A K28A mutant of MgTx was constructed by PCR mutagenesis using the primers 5′–GGTGCT**GCT**TGTATG-3 and 5′-CATACA**AGC**AGCACC-3′, where the underlined nucleotides denote the site of mutation.

PCR products were purified and cloned into the *Eco*R1 and *Sac*II sites of the yeast transfer vector pPICZαA. The recombinant plasmid was transformed into *E. coli* DH5α cells and transformants were selected using an agar plate with low salt LB (1% tryptone +0.5% yeast extract +0.5% NaCl +1.5% agar; pH 7.0) and 25 μg/ml of the antibiotic phleomycin D1 (Zeocin; Invitrogen). After the clone was confirmed by sequencing the insert, a total of 10 μg plasmid was linearized by digestion with *Sac*I. The linearized construct was transformed into *Pichia* strain X33 using heat shock (Pichia EasyComp; Invitrogen). Transformants integrated at the 5′-AOX1 locus using single crossover, and colonies were selected using an agar plate with yeast peptone dextrose (YPD) (1% yeast extract, 2% peptone, 2% glucose, and 2% agar) and 100 μg/ml zeocin. For each construct, 8–10 clones were initially chosen, and those found to produce the highest levels of MgTx and AgTx2 based on SDS-PAGE gel analysis of small-scale test expressions were selected for large-scale toxin expression.

We produced ^15^N-labelled MgTx and AgTx2 as follows: 100 μL of cell stock was grown at 30°C in 200 ml of ^15^N minimal medium (0.34% yeast nitrogen base (YNB) without ammonium sulfate and amino acids, 2% dextrose, 0.02% biotin, and 0.05% ^15^NH_4_Cl) in 100 mM potassium phosphate buffer at pH 6.0 with 100 μg/ml zeocin for 48 h. The cells were then transferred into 800 ml of ^15^N minimal medium. After another 48 h, the OD_600_ was 25–30. The cells were then centrifuged, collected, and grown in 1 litre of ^15^N minimal methanol medium (0.34% YNB without ammonium sulfate and amino acids, 0.02% biotin, 1% methanol, and 0.05% ^15^NH_4_Cl). Once every 24 h, 1% methanol was added to induce protein expression for 2 days. The supernatant was collected by centrifugation, dialyzed twice against 5 litres of H_2_O, then loaded onto a nickel affinity column equilibrated with Equilibration Buffer (25 mM Tris, 150 mM NaCl, pH 8.0). After washing with Equilibration Buffer to remove nonspecific binders, the recombinant toxin was eluted with Elution Buffer (25 mM Tris, 150 mM NaCl, 200 mM imidazole, pH 8.0). Recombinant MgTx and AgTx2 were further purified by reverse-phase (RP) HPLC. RP-HPLC was performed on a Vydac C_18_ column (250×4.6 mm; particle size 5 μm) using a flow rate of 1 ml/min and a gradient of 20–30% solvent B (0.1% trifluoroacetic acid in 90% acetonitrile) in solvent A (0.1% trifluoroacetic acid in water) over 40 min. The purity of the recombinant toxins was judged using tricine SDS-PAGE [26].

### Mass spectrometry

Matrix-assisted laser desorption/ionisation time-of-flight (MALDI-TOF) mass spectrometry was performed using a Model 4700 Proteomics Bioanalyser (Applied Biosystems, CA, USA). HPLC fractions were mixed (1∶1, v:v) with α-cyano-4-hydroxy-cinnamic acid matrix (10 mg/ml in 50% H_2_O/50% acetonitrile containing 0.1% formic acid) and MALDI-TOF spectra were collected in positive reflector mode. All masses given are for the monoisotopic M+H^+^ ions.

### Western blots

Proteins were separated under reducing conditions on a SDS-PAGE gel then transferred to a Hybond ECL nitrocellulose membrane (GE Healthcare Australia, NSW, Australia) by electroblotting at 400 mA for 1 h. The membrane was then washed several times with PBS and blocked with Odyssey blocking buffer for 1 h. Then membrane was treated with anti-His antibody (His-probe Antibody H-15 rabbit polyclonal IgG, Santa Cruz Biotechnology, CA, USA) for 60 min at room temperature. After 4–5 washes, the membrane was treated with secondary antibody (IRDye® 800CW goat polyclonal anti-rabbit IgG, LI-COR Biosciences, NE, USA) for 60 min at room temperature. The membrane was then washed 4–5 times with PBS plus Tween 20 followed by a final wash with PBS to remove the detergent. The membrane was then scanned using an Odyssey infrared imaging system (LI-COR Biosciences).

### Cells and cell lines

L929 mouse fibroblast cells stably expressing mouse K_V_1.1 or mouse K_V_1.3 channels [27] were kind gifts from Dr. George Chandy (University of California, Irvine). The cells were maintained in DMEM medium (Invitrogen, Carlsbad, CA, USA) supplemented with 10 IU/ml penicillin, 0.1 mg/ml streptomycin, 2 mM L-glutamine, 10% heat-inactivated fetal bovine serum, and 0.5 mg/ml G418 (EMD Chemicals, Gibbstown, NJ, USA). Freshly prepared buffy coats were purchased from the Gulf Coast Regional Blood Center (Houston, TX, USA) under a Baylor College of Medicine Institutional Review Board-approved protocol. Mononuclear cells were isolated from the buffy coats using Histopaque-1077 gradients (Sigma, St Louis, MI, USA) and used immediately or stored frozen in liquid nitrogen.

### Electrophysiology

Experiments were conducted at room temperature using the whole-cell patch-clamp technique, as described previously [4]. Patch pipettes had a resistance of 2–4 MΩ when filled with a solution containing (in mM): 145 KF, 10 HEPES, 10 EGTA, and 2 MgCl_2_, pH 7.2, 290 mOsm. The bath solution contained (in mM): 160 NaCl, 4.5 KCl, 2 CaCl_2_, 1 MgCl_2_, 10 HEPES, pH 7.2, 300 mOsm. K_V_ channel currents were elicited every 30 s by 200-ms depolarizing pulses from a holding potential of –80 mV to 40 mV. *K*_d_ values and Hill coefficients were determined by fitting the Hill equation to the reduction of peak current measured at 40 mV.

### Proliferation assays

Mononuclear cells (10^6^/ml) were plated into 96-well microplates and pre-incubated with peptidic K_V_1.3 blockers for 45 min at 37°C in RPMI medium (Invitrogen) supplemented with 100 IU/ml penicillin, 0.1 mg/ml streptomycin, 2 mM L-glutamine, 1 mM sodium pyruvate, 1% non-essential amino acids, 1% RPMI vitamins, 50 mM β-mercaptoethanol, and 1% heat-inactivated fetal bovine serum. T lymphocyte activation and proliferation was induced by the addition of 60 ng/ml anti-CD3 antibody (Clone OKT3, eBioscience, San Diego, CA, USA). Cells were cultured for 72 h at 37°C, 5% CO_2_, and [^3^H] thymidine was added during the last 16–18 h of culture. Cells were lysed by freezing at –20°C. After thawing, DNA was harvested onto fiberglass filters using a cell harvester (Inotech Biosystems International, Rockville, MD, USA). [^3^H] thymidine incorporation into the DNA of proliferating cells was measured using a β-scintillation counter (Beckman Coulter, Brea, CA, USA).

### NMR spectroscopy

NMR spectra were acquired at 600 MHz using a Bruker Avance 600 NMR spectrometer at National Cheng Kung University. Samples were prepared in 10% D_2_O/90% H_2_O or 100% D_2_O, then the pH was adjusted with KOD to 4.6. Data were processed using XWINNMR and analyzed with Aurelia software. 2D ^1^H NMR spectra were recorded in the phase-sensitive absorption mode with quadrature detection in both F1 and F2 dimensions [28]. Samples of 2 mM ^15^N labelled MgTx and AgTx2 were used to acquire 2D ^1^H-^15^N HSQC spectra as well as 3D ^15^N edited TOCSY-HSQC and NOESY-HSQC spectra. Mixing times of 30–90 ms and 60–150 ms were used for TOCSY and NOESY experiments, respectively. The observed ^1^H chemical shifts were referenced with respect to the H_2_O or HOD signal, which was taken as 4.754 ppm downfield from external sodium 3-trimethylsilylpropionate-2,2,3,3-d4 (TSP) in D_2_O (0.0 ppm) at 300 K. The nitrogen chemical shift was referenced to external ^15^NH_4_Cl (3 mM in 1 M HCl) at 300 K, which is at 24.93 ppm downfield from liquid NH_3_. ^1^H-^2^H exchange rates for backbone amide protons were measured by recording DQF-COSY spectra 24 h after dissolving the protein in 100% D_2_O [29].

^1^H and ^15^N resonance assignments have been deposited in the Biological Magnetic Resonance Bank (www.bmrb.wisc.edu) under accession numbers BMRB-15420 (rMgTx) and BMRB-15421 (rAgTx2).

## Results

### Expression, purification and characterization of MgTx and AgTx2

**Figure 2:**
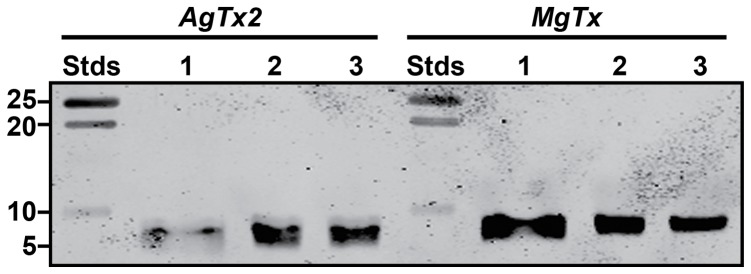
Western blot using anti-His_6_ primary antibody showing expression and purification of recombinant (A) AgTx2 and (B) MgTx produced in *P. pastoris*. The mass (in kDa) of the prestained molecular weight markers (“Stds”) is indicated. Lane 1, culture supernatant after methanol induction of toxin expression; Lane 2, recombinant peptide after purification using nickel affinity chromatography; Lane 3 recombinant peptide after RP-HPLC purification.

**Figure 3:**
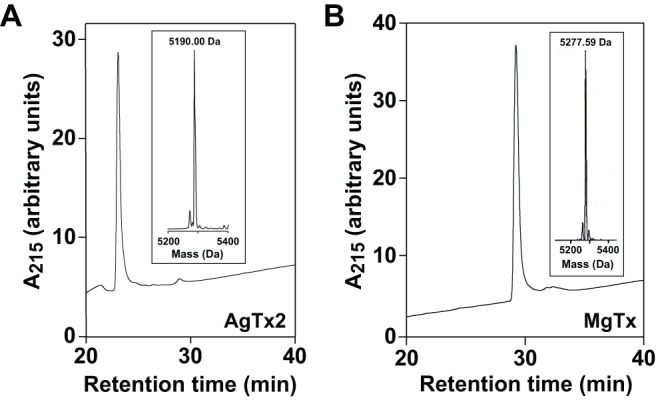
Chromatograms showing purification of (A) rAgTx2 and (B) rMgTx using HPLC. RP-HPLC was performed on a Vydac C_18_ column using a flow rate of 1 ml/min and a gradient of 20–30% acetonitrile over 40 min. The inset in each chromatogram is a MALDI-TOF spectrum showing the average mass of purified recombinant toxin.

AgTx2, MgTx, and MgTx-K28A were expressed using the pPICZαA vector in *P. pastoris* strain X-33. Each of the constructs encoded eight additional N-terminal amino acid residues (EFHHHHHH). The EF residues are vestiges of the vector, while the His_6_ tag was used for affinity purification of the toxins. Western blots summarising the overexpression and purification of AgTx2 and MgTx are shown in Fig. 2. Analysis of the blots revealed that the culture supernatants contained predominantly the soluble recombinant peptides (Fig. 2). The recombinant toxins were purified to homogeneity by nickel affinity chromatography followed by C18 RP-HPLC as shown in Fig. 3. The mass of both toxins estimated from the gel in Fig. 2 (∼7 kDa) was higher than expected, presumably because their high isoelectric point (pI ∼9.1 for both toxins) reduces their electrophoretic mobility. However, the masses of the purified recombinant proteins determined using mass spectrometry agreed well with the predicted masses for the fully oxidized peptides (MgTx: predicted  = 5278.1 Da, experimental  = 5277.6 Da; AgTx2 predicted  = 5190.0 Da, experimental  = 5190.0 Da; MgTx-K28A: predicted  = 5218.36 Da, experimental  = 5218.98). This indicated that, as expected, each of the recombinant toxins contains three disulfide bonds (Fig. 3). The yields of unlabelled rMgTx, rMgTx-K28A, and rAgTx2 produced in *P. pastoris* were 12–15 mg/L, 8–10 mg/mL, and 14–18 mg/L, respectively. The yields of ^15^N-labelled MgTx and AgTx2 obtained from expression of toxins in *P. pastoris* grown in ^15^N minimal medium were 5–7 mg/L and 6–8 mg/L, respectively, which is ∼50% less than the yields obtained from growth in rich medium.

#### Inhibition of mouse Kv1.3 and Kv1.1 by recombinant MgTx and AgTx2

**Figure 4:**
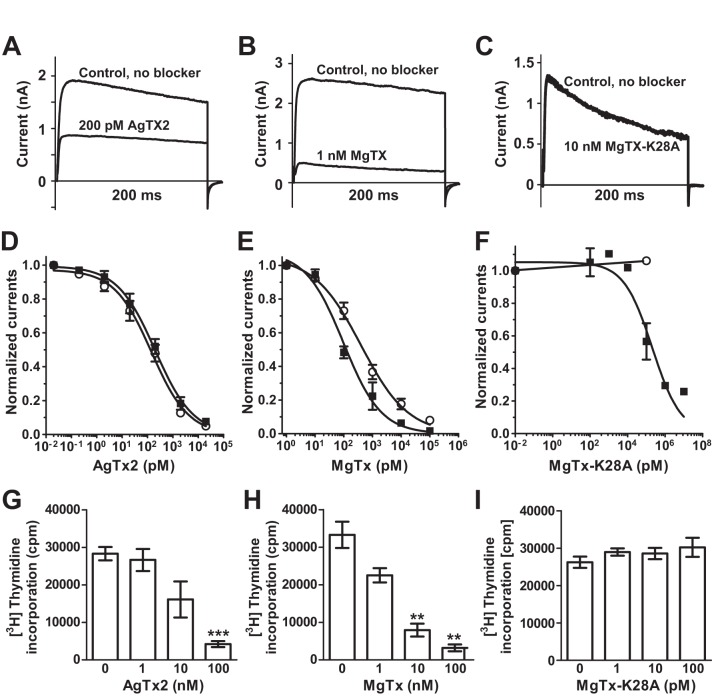
Inhibition of mouse K_V_ channels and human T lymphocyte proliferation by rAgTx2, rMgTx, and rMgTX-K28A. Inhibition of mK_V_1.3 currents in stably transfected L929 cells by (**A**) 200 nM rAgTX2, (**B**) 1 nM rMgTX, and (**C**) 10 nM MgTX-K28A. Dose-dependent inhibition of mK_V_1.3 (▪) and mK_V_1.1 (○) currents by (**D**) rAgTx2, (**E**) rMgTX, and (**F**) rMgTX-K28A (each data point is the mean of 3–5 determinations). Dose-dependent inhibition of [^3^H] thymidine incorporation by human T lymphocytes by (**G**) rAgTx2, (**H**) rMgTx, and (**I**) rMgTx-K28A. Error bars represent the SEM from three independent experiments with cells from different donors. Asterisks indicate statistically significant differences (***, p≤0.001; **, p<0.01).

AgTx2 and MgTx are reported to block K_V_1.1 and K_V_1.3 channels at picomolar concentrations [22], [30], [31]. We therefore measured the ability of rAgTx2 and rMgTx to block mouse K_V_1.3 (mK_V_1.3) and mouse K_V_1.1 (mK_V_1.1) channels stably expressed in L929 cells. As expected, rAgTx2 blocked both K_V_ currents with equivalent potency (IC_50_ = 201±39 pM on mK_V_1.3 and 144±30 pM on mKv1.1), with a Hill coefficient of 1 (Fig. 4A, D). rMgTx exhibited a higher affinity for mK_V_1.3 (IC_50_ = 97±3 pM) than for mK_V_1.1 (IC_50_ = 396±41 pM) (Fig. 4B, E). The recombinant rAgTx2 and rMgTx inhibited mK_V_1.3 with similar potency to the native toxins (200 pM and 30−100 pM for AgTx2 and MgTx, respectively; [31]).

We next tested the ability of each recombinant peptides to block the proliferation of freshly-isolated human T lymphocytes, which are sensitive to K_V_1.3 channel blockers [32]. Both rAgTx2 and MgTx inhibited the proliferation of T lymphocytes in a dose-dependent manner with an IC_50_ of ∼40 nM for rAgTx2 (Fig. 4G) and ∼6 nM for rMgTx (Fig. 4H). These concentrations are within the range previously described for MgTx (∼5 nM) and AgTx2 (>5 nM) [33], [34]. Thus the biological activities of rAgTx2 and rMgTx produced in *P.pastoris* are similar to the native toxins.

#### Production and characterization of rMgTx-K28A

α-KTx scorpion toxins that block K_V_ channels contain a conserved lysine residue that fits snugly into the pore of the channel and makes intimate contact with the selectivity filter [35]. Mutation of this residue (K27) in agitoxin causes a dramatic reduction in toxin binding and activity [36]. In order to test the utility of the *Pichia* system for producing toxin mutants, we decided to examine the effect of mutating the corresponding lysine residue (K28) in MgTx. The mutant toxin rMgTx-K28A was produced in high yield (8–10 mg/L). However, in contrast to rAgTx2 and rMgTx, rMgTx-K28A had no effect on mK_V_1.1 currents at concentrations up to 100 nM (Fig. 4F). The mutant toxin blocked mK_V_1.3 currents with an IC_50_ of 216±24 nM, representing a more than 2,000-fold loss in activity on this channel relative to the native toxin (Fig. 4C, F). Consistent with this markedly reduced activity on mK_V_1.3, the rMgTx-K28A mutant did not affect the proliferation of T lymphocytes (Fig. 4I).

#### Structural analysis of MgTx and AgTx2

**Figure 5:**
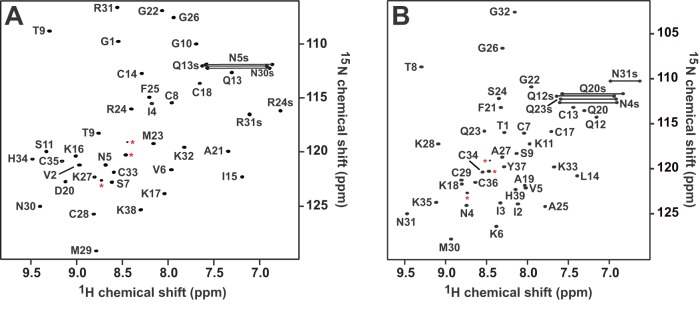
^1^H-^15^N HSQC spectra of recombinant (A) AgTx2 and (B) MgTx produced in *P. pastoris.* Correlation peaks are labelled according to residue type and sequence number. The peaks connected by horizontal lines correspond to the side-chain NH_2_ groups of Gln and Asn residues. Peaks marked with a red asterisk correspond to residues from the N-terminal His_6_ tag.

We acquired 2D and 3D NMR spectra of ^15^N-labelled rMgTx and rAgTx2 at the same pH as in previous studies [20], [21]. The ^1^H and ^15^N chemical shifts observed in the ^1^H-^15^N HSQC spectra of rMgTx and rAgTx2 are consistent with previously reported values (Fig. 5A, B). The ^1^H_N_ chemical shifts of the N-terminal Thr1 (Δδ  = −1.0 ppm) and Ile2 (Δδ  = −0.56 ppm) residues in MgTx, as well as Val2 (Δδ  = −0.15 ppm) in AgTx2, were shifted slightly upfield presumably due to the six extra N-terminal histidine residues. The similarity between the ^1^H_N_ and ^15^N chemical shifts we observed for recombinant MgTx and AgTx2 and those reported previously for native MgTx and AgTx2 are suggestive that the recombinant peptides have adopted the native fold. However, in order to confirm this, we performed additional experiments to determine the disulfide pairings and secondary structure of the recombinant toxins. We identified disulfide connectivities by searching for sets of Hβ to Hβ, Hβto Hα, and Hα to Hα NOEs between cysteine residues in a 3D NOESY-HSQC experiment acquired in 100% D_2_O [37]. Using this approach, we identified Cys7–Cys29, Cys13–Cys34 and Cys17–Cys36 disulfide bonds in rMgTx, and Cys8–Cys28, Cys14–Cys33 and Cys18–Cys35 disulfide bonds in rAgTx2. Thus, the disulfide-bond connectivities in the recombinant peptides produced in *P. pastoris* are identical to those in the native peptides [20], [21].

**Figure 6:**
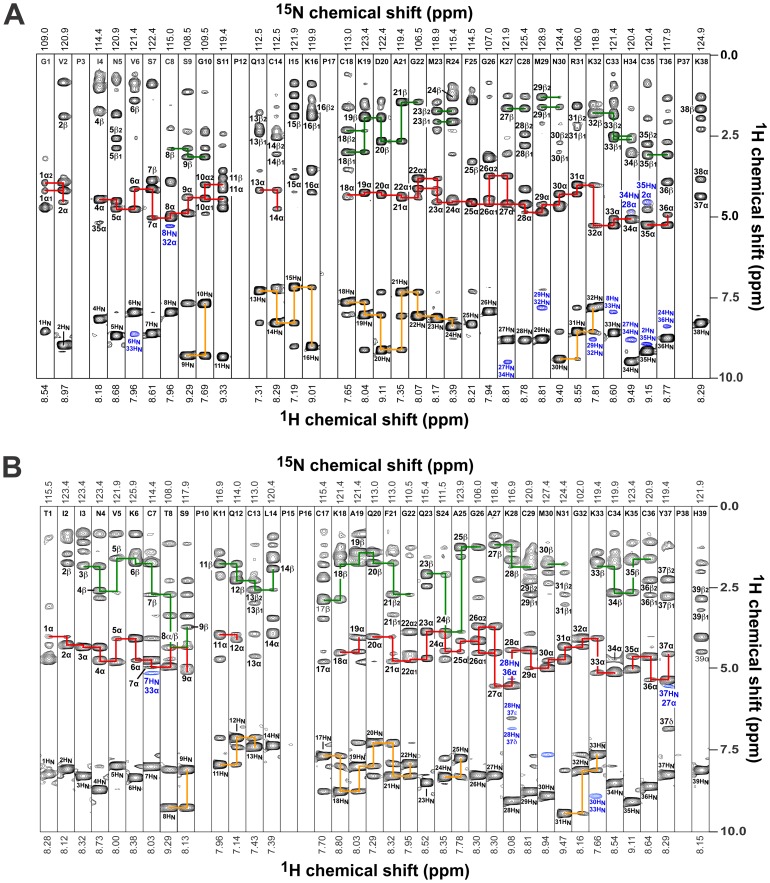
Amide-proton strips from 3D ^15^N-edited NOESY spectra of (A) rAgTx2 (G1–K38) and (B) rMgTx (T1–H39). Sequential αN, βN, and NN connectivities that facilitated sequence-specific resonance assignments are indicated by red, green, and orange lines, respectively. Medium- and long-range dipolar connectivities that facilitated assignment of secondary structure are highlighted and labelled in blue.

**Figure 7:**
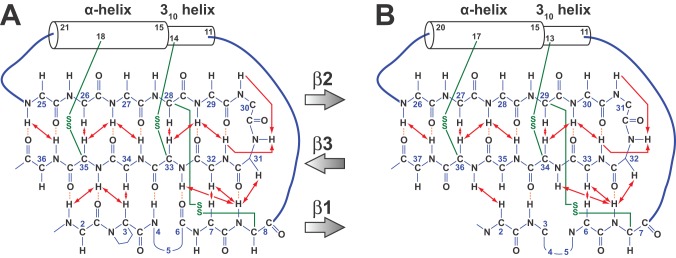
Secondary structure of recombinant (A) AgTx2 and (B) MgTx determined from NMR data. Dipolar connectivities observed in 2D NOESY spectra are shown as double-sided red arrows. Cross-strand hydrogen bonds inferred from ^1^H-^2^H exchange rates are depicted as dashed orange lines. Green lines indicate disulfide-bonds inferred from NOE connectivities. The shaded arrows indicate the direction (N→C) of the three β strands.

The secondary structure of rMgTx and rAgTx2 was determined by identification of NOE patterns characteristic of β-strands and α-helices in 2D and 3D NOESY spectra. The formation of antiparallel β-sheets was characterized by identification of H_α_-H_α_, H_α_-H_N_, and H_N_-H_N_ NOEs connecting strands (Fig. 6) and the presence of slowly exchanging amide protons. Strip plots from the ^15^N-edited NOESY-HSQC spectrum of MgTx (pH 4.6) clearly showed NOEs between H_α_ of Cys7 and H_N_ of Cys33; H_α_ of Lys28 and H_N_ of Tyr37; H_α_ of Tyr37 and H_N_ of Lys28; and between H_N_ of Met30 and H_N_ of Lys33 (Fig. 6A). Similarly, strip plots from the ^15^N-edited NOESY-HSQC spectrum of AgTx2 (pH 4.6) revealed NOEs between H_α_ of Cys8 and H_N_ of Cys33; H_α_ of Lys27 and H_N_ of Thr36; H_α_ of Thr36 and H_N_ of Lys27; H_N_ of Val2 and H_N_ of Cys35; H_N_ of Val6 and H_N_ of Cys33; H_N_ of Arg24 and H_N_ of Thr36; H_N_ of Met29 and H_N_ of Lys32; and between H_N_ of Lys27 and H_N_ of His34 (Fig. 6B). Furthermore, non-labile backbone amide protons were identified in both toxins by their slow exchange rate in ^1^H/^2^H exchange experiments. Based on the identification of slowly exchanging backbone amide protons and the dipolar connectivities shown in Fig. 6, we were able to confirm the formation of triple stranded antiparallel β-sheets in each toxin (Fig. 7A, B). Thus, the combined NMR data reveals that the recombinant AgTx2 and MgTx produced in *P. pastoris* adopt the same 3D fold as the native toxins.

## Discussion

### K_V_1.3 as a therapeutic target

K_V_ channels have diverse physiological functions; they play important roles in cell excitability, muscle contraction, regulation of neuronal and cardiac electrical functions, and propagation of action potentials [1]. The K_V_1.3 subtype is upregulated during activation of terminally differentiated human effector memory T cells, which are important mediators of autoimmune diseases such as MS, T1D, and rheumatoid arthritis [3]–[6]. This has led to the hypothesis that compounds that selectively target K_V_1.3 channels could be used to treat autoimmune diseases [38]. Consistent with this idea, ShK, a sea anemone toxin that potently blocks K_V_1.3, reverses symptoms of experimental autoimmune encephalomyelitis and suppresses delayed type hypersensitivity in rats [3]. A stabilized version of ShK is about to enter Phase 1 clinical trials for the treatment of multiple sclerosis [39]. Thus, there is considerable interest in characterizing other blockers of K_V_1.3 and determining their molecular mechanism of action in order to facilitate the rational design of small-molecule blockers of this channel.

Many animal toxins have extremely high specificity and potency for their molecular target due to millions of years of evolutionary fine-tuning. These features have made animal toxins extremely valuable as pharmacological tools and as leads for the development of novel therapeutics [10], [11], [40]. Scorpions are one of the best studied venomous animals. Along with centipedes, they are the oldest venomous animals, with the earliest scorpion fossils dating back to the Silurian period ∼430 Mya [41]. The predominant components of scorpion venoms are disulfide-rich peptides, most of which modulate the activity of a wide variety of ion channels, including K_V_ channels. 18 families of peptidic K_V_ blockers, comprising 75 different peptides, have been described from scorpion venoms [42]. These peptides, known as α-KTx toxins, are comprised of 20–40 amino acid residues and 3–4 disulfide bonds.

Some α-KTxs are of significant interest because they potently block the human K_V_1.3 channel. For example, both MgTx (α-KTx 2.2) and AgTx2 (α-KTx 3.2) potently inhibit K_V_1.3 by blocking the central ion-conducting pore [21], [22], [43]. However, despite their similar 3D fold and high sequence homology, the affinity of α-KTx toxins for K_V_1.3 varies over six orders of magnitude from picomolar to micromolar. Moreover, many of these toxins inhibit a number of different subtypes K_V_ channels with high affinity, an undesirable attribute for peptides being considered for therapeutic use due to the potential for deleterious side-effects. Selectivity can be improved by engineering the peptides through truncation of specified regions or by mutating particular amino acid residues. For example, ADWX-1, a mutant of BmKTX (α-KTx 3.6) with three residue changes, exhibits 80-fold higher affinity towards K_V_1.3 (IC_50_ 1.89 pM) than the native peptide (IC_50_ of 150 pM) [44]. Clearly, a system that allowed efficient production of toxin analogs in order to develop structure-activity relationships (SARs) would facilitate future engineering efforts aimed at improving potency and selectivity against K_V_1.3.

### Development of a Pichia expression system for production of K_v_1.3 blockers

There are three major strategies for obtaining venom peptides: classical biochemical isolation from venom, chemical synthesis, and recombinant production in a heterologous system [19]. Since most venomous animals are very small, bioassay-guided isolation from venom usually produces only a limited amount of native peptide, thus precluding detailed SAR studies. Since most of these peptides contain 35–70 amino acid residues that are connected by 3–4 disulfide bridges, chemical synthesis of these peptides can be challenging both in terms of synthetic efficiency and the need to oxidatively refold the synthetic material to produce the correct disulfide-bond isomer. In some cases, the synthetic efficiency can be improved by splitting the synthesis into smaller fragments that are subsequently joined by native chemical ligation [45]. However, chemical synthesis is expensive, especially if one wishes to incorporate stable isotopes for NMR studies.

Recombinant production is the most cost-effective method for producing disulfide-rich peptides. The most common host for heterologous expression of venom peptides is *E. coli* [46], [47] but this often necessitates oxidative refolding of the peptides [48] as the redox environment of the *E. coli* cytoplasm is not favorable for disulfide-bond formation [16]. Higher yields of correctly folded toxin can often be obtained by exporting the peptides into periplasm of *E. coli*, where the cellular machinery for disulfide-bond formation is located [17]–[19]; however, the overall yield of recombinant peptide is typically less than 5 mg/liter. Recombinant expression of disulfide-rich peptides in eukaryotic expression systems has also been reported. For example, the spider-venom peptide PcTx1, a potent blocker of acid sensing ion channel 1a, was successfully produced in insect cell lines, albeit at a very low yield of 0.5 mg/liter [49].

An alternative host for heterologous expression of disulfide-rich venom peptides is *P. pastoris*, which offers several advantages compared to other expression systems: (i) *P. pastoris* grows faster to reach higher cell densities in simpler media; (ii) *Pichia* grows well in minimal medium, making it suitable for production of isotopically labelled peptides; (iii) expressed peptides can be secreted into the culture medium, which simplifies purification as *Pichia* secretes low levels of endogenous proteins [23]–[25]. *Pichia* has proved to be an excellent system for production of disulfide-rich snake-venom peptides, achieving yields as high as 150 mg/liter [24]. Thus, we attempted to develop a *Pichia* expression system for efficient production of disulfide-rich K_V_1.3 blockers, using AgTx2 and MgTx as prototypic examples.

Previously, both MgTx and AgTx2 were expressed in *E. coli* at final yields of 2–3 mg/L [21], [22]. In the present study, using the *Pichia* expression system, we were able to produce these toxins with a 6-fold higher yield of 12–18 mg/liter. Growth of cells in minimal medium in order to produce ^15^N-labelled rAgTx2 and rMgTx for NMR studies still produced an acceptable yield of 5–7 mg protein/liter of culture. The recombinant toxins not only retained their native fold but blocked mouse K_V_1.3 at picomolar concentrations and had the same selectivity for K_V_1.3 over K_V_1.1 as the native peptides. Moreover, both recombinant peptides potently inhibited the proliferation of human T lymphocytes. The IC_50_ values for inhibition of mouse K_V_1.3 are slightly higher than for the native toxins, presumably due to the presence of an N-terminal His_6_ tag. This tag could be easily removed by re-engineering the expression plasmids to incorporate a protease cleavage site immediately before the N-terminal residue of the peptide.

Finally, we demonstrated the utility of the *Pichia* expression system for producing toxin mutants in order to study structure-function relationships. Recent docking studies predicted that residue K28 in MgTx, which is conserved in α-KTx scorpion toxins, inserts into the pore of the K_V_1.3 channel and makes contact with the channel's selectivity filter [50]. In support of this prediction, we found that mutation of MgTx residue K28 to alanine completely abolished the ability of the toxin to inhibit mK_V_1.1 and reduced its activity on mK_V_1.3 by more than 2000-fold. Consistent with its markedly reduced activity on mKv1.3, the mutant toxin was unable to inhibit T lymphocyte proliferation.

In summary, we have developed an efficient *Pichia* expression system for production of K_V_1.3 blockers. The two prototypic K_V_1.3 blockers we produced using this system were obtained in high yield, and the recombinant toxins were properly folded and fully functional. This expression system will enable production of larger quantities of these toxins for probing the role of K_V_1.3 in autoimmune diseases, and it will also facilitate the production of toxin mutants for structure-function studies. Moreover, the heterologous expression system described here should be suitable for the production of not only peptidic K_V_1.3 blockers but also other venom peptides with multiple disulfide bonds.
